# Association between dexmedetomidine administration and 28-day mortality in critically ill patients with ventilator-associated pneumonia

**DOI:** 10.3389/fphar.2026.1785115

**Published:** 2026-06-25

**Authors:** Songmei Guan, Congcong Lv, Shigang Duan, Jianmin Qu, Tingting Wang

**Affiliations:** 1 Department of Pharmacy, Zhejiang Provincial People’s Hospital Bijie Hospital (The First People’s Hospital of Bijie), Bijie, Guizhou, China; 2 Hematopoietic Stem Cell Transplantation Center, State Key Laboratory of Experimental Hematology, Haihe Laboratory of Cell Ecosystem, National Clinical Research Center for Blood Diseases, Institute of Hematology and Blood Diseases Hospital, Chinese Academy of Medical Sciences and Peking Union Medical College, Tianjin, China; 3 Tianjin Institutes of Health Science, Tianjin, China; 4 Hepatobiliary and Pancreatic Surgery, Zhejiang Provincial People’s Hospital Bijie Hospital (The First People’s Hospital of Bijie), Bijie, Guizhou, China; 5 Department of Intensive Care Unit, Tongxiang First People’s Hospital, Tongxiang, Zhejiang, China; 6 Department of Intensive Care Unit, The Second People’s Hospital of Liaocheng, Linqing, Shandong, China

**Keywords:** dexmedetomidine, intensive care unit, MIMIC-IV database, mortality, ventilator-associated pneumonia

## Abstract

**Background:**

Ventilator-associated pneumonia (VAP) is a frequent intensive care unit (ICU) complication linked to prolonged ventilation, extended ICU and hospital stays, increased costs, and higher mortality. Current guidelines recommend light sedation with dexmedetomidine (DEX) or propofol for ventilated adults. Although DEX has shown anti-inflammatory effects in animal models, its impact on VAP outcomes remains unclear. This study aims to investigate the association between DEX administration and 28-day mortality in patients with VAP.

**Methods:**

This retrospective cohort study enrolled VAP patients from the Medical Information Mart for Intensive Care (MIMIC) (version 3.0) database, categorized into DEX-treated and non-DEX groups during ICU admission. The primary outcome was 28-day mortality. Secondary outcomes included ICU, in-hospital mortality. Kaplan-Meier (KM) survival curves with log-rank tests and Cox proportional hazards models evaluated the association between DEX use and mortality outcomes. To adjust for baseline differences, 1:1 propensity score matching (PSM) was applied. Interaction and subgroup analyses further examined result consistency.

**Results:**

Data from 1,753 patients were analyzed, comprised 901 in the DEX group and 852 in the non-DEX group. The analysis of KM curves revealed a significantly lower 28-day mortality rate in the DEX group compared to the non-DEX group. After adjusting for multiple confounding factors, the Cox regression model demonstrated a significant protective impact of DEX-use on the risk of 28-day mortality in patients with VAP, with hazard ratios (HR) of 0.54 (95% confidence interval (CI): 0.43–0.67, p < 0.001). PSM analysis confirmed these results, showing HR of 0.81 (95% CI: 0.65–0.99, p = 0.044). No interactions were observed between DEX-use and 28-day mortality across stratified variables.

**Conclusion:**

DEX administration during the ICU stays was associated with improved outcomes in critically ill patients with VAP. The results need to be verified in randomized controlled trials.

## Introduction

Ventilator-associated pneumonia (VAP) remains one of the most prevalent hospital-acquired lower respiratory tract infections in intensive care unit (ICU) worldwide, affecting 5%–40% of critically ill patients undergoing mechanical ventilation (MV) (Johnstone et al., 2023). Its incidence, estimated at 2 to 30 cases per 1,000 ventilator days depending on definitions and diagnostic methodologies, underscores the substantial burden VAP imposes on healthcare systems globally (Ego et al., 2015; Metersky et al., 2016). The process of MV itself compromises the airway’s physical barriers, providing a direct entry point for pathogens, reducing immune defenses, and increasing infection risk (Karakuzu et al., 2018). Pulmonary infections in critically ill patients may precipitate septic shock, acute respiratory distress syndrome (ARDS), or both, with mortality rates ranging from 30% to 50% (Pirracchio et al., 2024).

Sedation is a cornerstone of ICU care for mechanically ventilated patients, alleviating discomfort, reducing stress responses, and improving ventilation effectiveness. The 2013 Pain, Agitation, and Delirium (PAD) guidelines conditionally recommend non-benzodiazepine sedatives, such as dexmedetomidine (DEX) or propofol, over benzodiazepines like midazolam or lorazepam for mechanically ventilated critically ill patients. This preference is based on evidence linking non-benzodiazepine sedatives to improved short-term outcomes, including shorter ICU stays, reduced duration of mechanical ventilation, and lower delirium incidence (Barr et al., 2013; Devlin et al., 2018).

DEX, a highly selective α_2_-adrenergic receptor agonist, is increasingly employed as an alternative sedative due to its ability to provide lighter sedation, reduce delirium, offer analgesia, and decrease opioid requirements in postoperative patients (Yuan et al., 2020; Lewis et al., 2022). By activating α2-adrenergic receptors, DEX inhibits thyroxine release, thereby attenuating sympathetic nervous system activity (Weerink et al., 2017). Additionally, DEX avoids respiratory depression, making it particularly advantageous for patients who experience difficulties in weaning from mechanical ventilation (Su et al., 2016; Lewis et al., 2022). DEX has also shown potential benefits in improving hemodynamic stability in postoperative patients, including reducing inotropic requirements and exerting antiarrhythmic effects (Wadile et al., 2023; Peng et al., 2024), making it an appealing choice for sedation after congenital heart surgery. Additionally, dexmedetomidine exhibits significant anti-inflammatory properties (Taniguchi et al., 2004; Xu et al., 2013; Li et al., 2015). Experimental and clinical studies have demonstrated its ability to suppress pro-inflammatory cytokines such as IL-6 and TNF-α (Fujimoto et al., 2021). Recent findings further reveal that dexmedetomidine alleviates LPS-induced lung inflammation in septic mice via the α7nAChR-dependent cholinergic anti-inflammatory pathway (Liu et al., 2016). However, whether these anti-inflammatory effects translate into improved outcomes in patients with sepsis, particularly those with VAP, remains uncertain.

Given the high morbidity and mortality of VAP, there is an urgent need to evaluate therapies that may improve outcomes. While DEX has shown promise in various clinical settings, its impact on VAP management, particularly survival, remains unclear. This study aims to assess the relationship between DEX administration and 28-day mortality in critically ill patients with VAP using a large retrospective dataset. The results may offer valuable insights into optimizing sedation strategies and improving outcomes for this at-risk population.

## Methods

### Study design

We conducted a retrospective cohort study using data from the MIMIC-IV database (version 3.0), released on 6 January 2023. This database contains deidentified clinical information from 94,458 ICU patients admitted to Beth Israel Deaconess Medical Center between 2008 and 2022 (Johnson et al., 2023). This database was used with permission obtained by Jianmin Qu, one of the authors (certificate ID 31797517). The study was approved by the Human Research Ethics Committee of The Second People’s Hospital of Liaocheng (Approval No. 2025-043), with a waiver of informed consent due to retrospective nature of the study. The study was reported in accordance with the RECORD (Reporting of studies Conducted using Observational Routinely-collected health Data) Statement (Benchimol et al., 2015).

### Participants

Our study population comprised adults (≥18 years) with a first ICU admission who carried a discharge diagnosis of VAP, as defined by the International Classification of Diseases, Ninth Revision (ICD-9) codes 4957 and 99731, and 10th Revision (ICD-10) code J95851. Patients with multiple ICU admissions during the same hospitalization or across different hospitalizations were excluded, and only the first ICU admission was considered for analysis to ensure statistical independence and population homogeneity. Patients with an ICU stay of less than 24 h and who were pregnant were excluded. All eligible patients meeting the predefined inclusion and exclusion criteria were extracted from the database; no additional sampling procedure was applied. Eligible patients were divided into two groups: those who received DEX during their ICU stay and those who did not (non-DEX group).

### Data extraction

Patient data were extracted from the database using Structured Query Language (SQL) by two authors (Guan and Qu). Following extraction, the data were independently reviewed by another author (Wang) to ensure accuracy and completeness. All authors with database access completed the required CITI program certification for MIMIC database usage. The following clinical information was collected for each patient: demographics, laboratory measurements, vital signs, comorbidities, severity of illness, and interventions. Laboratory parameters and vital signs were obtained from the first 24 h of ICU admission. For variables with multiple measurements, we selected the value indicating the greatest severity of illness (e.g., highest for heart rate, lactate; lowest for mean arterial pressure (MAP), hemoglobin, potential of hydrogen (pH), as detailed in Supplementary Table 1. Comorbidities included conditions such as diabetes mellitus (DM), hypertension, chronic pulmonary disease (CPD), myocardial infarct (MI), congestive heart failure (CHF) and Sepsis-3 (Singer et al., 2016). The severity of illness at admission was assessed using acute physiology and chronic health evaluation (APACHE II) (Hu et al., 2023), simplified acute physiologic score (SAPS) II (Sánchez-Díaz et al., 2022) and modified nutrition risk in critically ill (mNUTRIC) (Tseng et al., 2021). Interventions within the first 24 h of ICU admission were recorded, including mechanical ventilation (MV), vasoactive drugs (VA), renal replacement treatment (RRT), antibiotics and sedative-analgesic medications (Table 1). Covariates were chosen based on prior literature and clinical experience (Hu et al., 2022; Zhang et al., 2022). The study protocol was developed based on clinical knowledge and prior literature, with a predefined list of variables and extraction rules. After data extraction, we performed comprehensive data cleaning and quality checks, including assessment of missing data patterns, identification of outliers, and verification of variable distributions, to ensure the robustness of the subsequent analyses. Multicollinearity was assessed by calculating the variance inflation factor (VIF) for each variable as detailed in Supplementary Table 2. A VIF >5 was considered indicative of problematic multicollinearity, while a VIF >10 suggested severe multicollinearity (Hu et al., 2023).

**TABLE 1:** Baseline characteristics of the two groups before and after PSM.

Variable	Unmatched patients		Matched patients			
	DEX (non-use)	DEX (use)	SMD	DEX (non-use)	DEX (use)	SMD
N	852	901		601	601	
General characteristics						
Age, (years)	65.13 (15.62)	60.17 (16.12)	0.313	62.86 (16.20)	63.80 (15.43)	0.060
Gender, (female), n (%)	320 (37.60)	273 (30.30)	0.154	197 (32.80)	197 (32.80)	<0.001
Race, (non-White), n (%)	394 (46.20)	471 (52.30)	0.121	297 (49.40)	300 (49.9)	0.010
BMI, (kg/m^2^)	29.23 (8.04)	31.02 (9.37)	0.204	30.08 (8.41)	30.03 (8.69)	0.006
Vital signs						
Heart rate, (bpm)	106.46 (21.11)	107.97 (21.48)	0.071	106.66 (20.81)	106.63 (21.33)	0.002
Respiratory rate, (bpm)	28.29 (6.34)	29.68 (7.14)	0.206	28.93 (6.53)	28.98 (6.57)	0.008
MAP, (mmHg)	58.25 (14.36)	57.92 (14.07)	0.023	57.88 (14.39)	58.22 (13.61)	0.024
Laboratory parameters						
WBC, (10^9^/L)	15.57 (11.92)	15.94 (7.93)	0.037	15.95 (13.29)	15.71 (7.85)	0.022
HB, (g/dL)	10.33 (2.43)	10.40 (2.55)	0.027	10.31 (2.51)	10.31 (2.55)	0.001
PLT, (10^9^/L)	184.65 (100.38)	186.52 (97.01)	0.019	179.31 (97.69)	179.86 (94.43)	0.006
AG, (mEq/L)	16.87 (5.28)	16.76 (5.11)	0.022	16.78 (5.21)	17.17 (5.18)	0.076
Bicarbonate, (mEq/L)	21.19 (5.33)	20.74 (5.26)	0.085	20.79 (5.18)	20.59 (5.23)	0.040
Scr, (mg/dL)	1.57 (1.54)	1.83 (1.73)	0.156	1.70 (1.69)	1.81 (1.64)	0.071
ALT, (IU/L)	149.78 (568.66)	194.02 (1003.59)	0.054	180.07 (658.19)	153.06 (545.63)	0.045
Sodium, (mEq/L)	140.84 (5.94)	140.92 (5.73)	0.013	140.92 (5.88)	140.97 (6.08)	0.009
Calcium, (mg/dL)	7.97 (0.95)	7.93 (0.89)	0.035	7.92 (0.95)	7.97 (0.88)	0.055
Potassium, (mEq/L)	3.82 (0.62)	3.92 (0.58)	0.176	3.86 (0.63)	3.85 (0.57)	0.018
PaCO_2_, (mmHg)	49.00 (15.90)	51.01 (14.75)	0.131	49.77 (15.24)	49.40 (13.47)	0.026
PFR, (mmHg)	208.72 (123.43)	175.94 (114.13)	0.276	189.03 (111.72)	188.49 (120.50)	0.005
pH	7.31 (0.12)	7.29 (0.11)	0.212	7.30 (0.12)	7.29 (0.11)	0.015
Lactate, (mmol/L)	2.88 (2.87)	3.06 (2.81)	0.062	3.10 (3.11)	3.11 (2.92)	0.001
APTT, (s)	48.06 (34.78)	47.04 (33.65)	0.030	48.87 (35.55)	48.72 (35.64)	0.004
Disease severity scores						
APACHE II	20.39 (7.77)	20.86 (8.38)	0.059	20.80 (8.14)	21.27 (8.53)	0.056
SOFA	6.22 (3.99)	6.89 (3.85)	0.173	6.71 (4.10)	6.76 (4.02)	0.012
CCI	5.90 (2.74)	5.17 (3.04)	0.252	5.60 (2.74)	5.85 (3.06)	0.083
mNUTRIC	4.29 (1.97)	4.21 (2.11)	0.042	4.31 (2.03)	4.43 (2.12)	0.061
Comorbidities						
MI, n (%)	149 (17.5)	162 (18.0)	0.013	109 (18.1)	117 (19.5)	0.034
CHF, n (%)	261 (30.6)	256 (28.4)	0.049	186 (30.9)	191 (31.8)	0.018
CPD, n (%)	222 (26.1)	228 (25.3)	0.017	156 (26.0)	153 (25.5)	0.011
Hypertension, n (%)	544 (63.8)	566 (62.8)	0.021	382 (63.6)	385 (64.1)	0.010
DM, n (%)	244 (28.6)	280 (31.1)	0.053	189 (31.4)	184 (30.6)	0.018
Sepsis, n (%)	779 (91.4)	879 (97.6)	0.271	582 (96.8)	579 (96.3)	0.028
Septic shock, n (%)	198 (23.2)	295 (32.7)	0.213	162 (27.0)	192 (31.9)	0.110
Medication or procedures						
Fentanyl, n (%)	508 (59.6)	687 (76.2)	0.362	417 (69.4)	407 (67.7)	0.036
Midazolam, n (%)	263 (30.9)	281 (31.2)	0.007	200 (33.3)	186 (30.9)	0.050
Morphine, n (%)	113 (13.3)	58 (6.4)	0.231	56 (9.3)	54 (9.0)	0.012
Propofol, n (%)	505 (59.3)	701 (77.8)	0.407	422 (70.2)	420 (69.9)	0.007
Antibiotic, n (%)	519 (60.9)	626 (69.5)	0.180	394 (65.6)	397 (66.1)	0.011
VA, n (%)	375 (44.0)	443 (49.2)	0.103	283 (47.1)	278 (46.3)	0.017
MV, n (%)	688 (80.8)	778 (86.3)	0.151	511 (85.0)	498 (82.9)	0.059
RRT, n (%)	22 (2.6)	50 (5.5)	0.151	21 (3.5)	32 (5.3)	0.089

Medication or procedures refer to those used on the first day of ICU admission and all of the continuous variables are presented as mean (SD).

Abbreviations: PSM: propensity score matching, DEX: dexmedetomidine, SMD: standardized mean differences, BMI: body mass index, MAP: mean arterial pressure, WBC: white blood cell, HB: hemoglobin, PLT: platelet, AG: anion gap, Scr: serum creatinine, ALT: alanine aminotransferase, PaCO_2_: partial pressure of carbon dioxide in arterial blood, PFR: PaO_2_/FiO_2_ ratio, pH: potential of hydrogen, APTT: activated partial thromboplastin time, APACHE II: Acute Physiology and Chronic Health Evaluation II, SOFA: sequential organ failure assessment, CCI: Charlson Comorbidity Index, mNUTRIC: Modified Nutrition Risk in Critically ill, MI: myocardial infarction, CHF: congestive heart failure, CPD: chronic pulmonary disease, DM: diabetes mellitus, VA: vasopressor agent, MV: mechanical ventilation, RRT: renal replacement treatment.

### Outcomes

28-day mortality was defined as death from any cause within 28 days starting from admission to the ICU. The primary outcome of this study was 28-day all-cause mortality. Secondary outcomes included in-hospital mortality and ICU mortality.

### Statistical analysis

In this study, with the exception of alanine aminotransferase (ALT) and lactate, the percentage of missing data for all other covariates was below 20%. Detailed information is provided in Supplementary Table 3. Multiple imputation for all missing variables was conducted using the mice package in R (R Foundation for Statistical Computing) (The R Project for Statistical Computing, 2026). Baseline patient characteristics were stratified across different groups (Table 1). Categorical variables are presented as numbers (percentages), while continuous variables are expressed as mean ± standard deviation or median (interquartile range), depending on their distribution. Group comparisons between the DEX and non-DEX cohorts were conducted using independent t-tests or Mann–Whitney U tests for continuous variables, and chi-squared tests for categorical variables.

In our study, PSM was applied with a caliper value of 0.2 to minimize baseline differences between the two groups. To assess the effectiveness of PSM in balancing covariates, we calculated the standardized mean difference (SMD) for each variable. An SMD greater than 0.1 was considered indicative of a meaningful imbalance in covariate prevalence (Pham et al., 2020).

Cox proportional hazards models were applied to estimate the HRs and 95% CIs for the association between DEX use and clinical endpoints. Both unadjusted and adjusted models were analyzed as follows: Model 1 examines the unadjusted association between dexmedetomidine use and mortality. Model 2 adjusts for demographic characteristics such as age, sex, race, and BMI. Model 3 further incorporates physiological parameters measured within the first 24 hours of ICU admission—including vital signs (e.g., HR, MAP), laboratory values (e.g., SCr, lactate), and acid-base status (e.g., pH, bicarbonate). Model 4 provides the fully adjusted analysis, adding clinical severity scores (APACHE II, SOFA), relevant comorbidities (e.g., DM, CPD), and concurrent treatments (e.g., VA, MV, and other sedatives). This stepwise approach allows us to progressively account for potential confounders and isolate the independent effect of dexmedetomidine.

Kaplan-Meier survival curves were used to depict 28-day survival. The unadjusted curves were compared using the log-rank test, while the differences in the covariate-adjusted curves (Model 4) were assessed via the likelihood ratio test.

Subgroup analysis was conducted based on age (<60 years and ≥60 years), SOFA (<5 and ≥5), gender, comorbidities including CPD, DM, septic shock, CHF. For each subgroup, HRs and 95% CIs were calculated.

Sensitivity analyses were conducted to evaluate the robustness of our findings and the influence of different association inference models on the conclusions. Specifically, we employed additional models, including propensity-score adjusted (PSA) (Shin et al., 2022), PSM (Austin, 2011), inverse probability of treatment weighting (IPTW) (Austin and Stuart, 2015), standardized mortality ratio weighting (SMRW) (Brookhart et al., 2013), pairwise algorithmic (PA) (Li and Greene, 2013) and overlap weight (OW) (Cheng et al., 2022). Effect sizes and corresponding p-values from these models were calculated, reported, and compared to assess consistency.

As the sample size was determined by the availability of data, no prior statistical power calculation was performed. Data analysis was conducted using R software and Free Statistics software (Wang et al., 2024). A P-value of <0.05 was considered statistically significant.

## Results

### Patient characteristics

**FIGURE 1:**
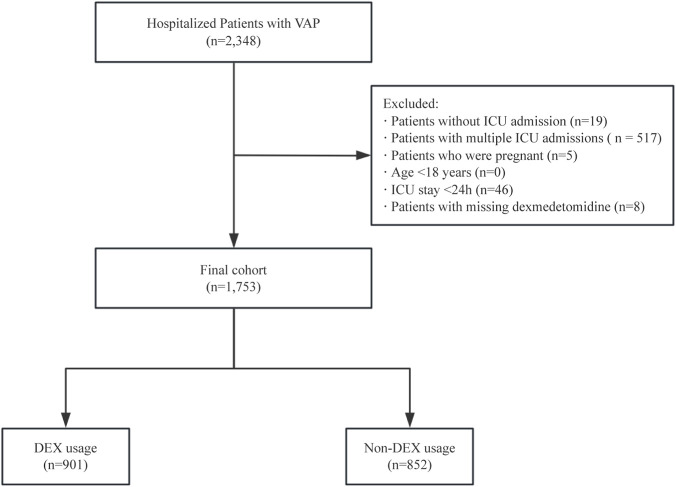
Flowchart of patient selection.

The study included 1,753 critically ill patients with VAP, consisting of 901 patients in the DEX group and 852 in the non-DEX group (Figure 1). After PSM, 601 patients who received DEX were matched with 601 patients who did not. Baseline characteristic analysis revealed significant differences between the two groups. Patients in the DEX group were older, predominantly male, and presented with more severe conditions, including higher levels of SCr and SOFA score as well as lower PFR. The DEX group also had more frequent use of medications such as antibiotics, fentanyl, and propofol, higher rates of sepsis, and greater reliance on life support measures, including MV, RRT and VA. Following PSM, the two groups achieved excellent baseline balance, with all SMDs below 0.1, as detailed in Table 1.

### Outcomes

The 28-day mortality rates were 17.1% (154/901) in the DEX group and 27.3% (233/852) in the non-DEX group, indicating significantly lower mortality in the DEX group. Similarly, the DEX group showed a reduced risk of ICU mortality in both the unadjusted model (Model 1: HR, 0.46 [95% CI: 0.37–0.58], p < 0.001) and the fully adjusted model (Model 4: HR, 0.40 [95% CI: 0.31–0.52], p < 0.001). Furthermore, patients in the DEX group had better overall survival outcomes, including lower in-hospital mortality (18.8% vs. 29.8%) and ICU mortality (14.4% vs. 20.1%) compared to the non-DEX group (Table 2).

**TABLE 2:** The associations of DEX use and outcomes in patients with VAP.

Variable	Total, n	Event, n(%)	Model 1	Model 2	Model 3	Model 4
			HR (95% CI)	HR (95% CI)	HR (95% CI)	HR (95% CI)
28-day mortality						
DEX (non-use)	852	233 (27.3)	1(Ref)	1(Ref)	1(Ref)	1(Ref)
DEX (use)	901	154 (17.1)	0.57 (0.47∼0.70)	0.65 (0.53∼0.80)	0.61 (0.49∼0.75)	0.54 (0.43∼0.67)
*P* value			<0.001	<0.001	<0.001	<0.001
In-hospital mortality						
DEX (non-use)	852	254 (29.8)	1(Ref)	1(Ref)	1(Ref)	1(Ref)
DEX (use)	901	169 (18.8)	0.52 (0.43∼0.63)	0.58 (0.47∼0.70)	0.56 (0.45∼0.68)	0.49 (0.39∼0.60)
*P* value			<0.001	<0.001	<0.001	<0.001
ICU mortality						
DEX (non-use)	852	171 (20.1)	1(Ref)	1(Ref)	1(Ref)	1(Ref)
DEX (use)	901	130 (14.4)	0.46 (0.37∼0.58)	0.48 (0.38∼0.61)	0.57 (0.47∼0.70)	0.40 (0.31∼0.52)
*P* value			<0.001	<0.001	<0.001	<0.001

Model 1: Unadjusted; Model 2: Adjusted for gender, age, race, BMI; Model 3: Model 2 plus heart rate, respiratory rate, MAP, HB, PLT, WBC, AG, Scr, ALT, APTT, potassium, sodium, calcium, pH, PaCO_2_, PFR, lactate, bicarbonate; Model 4: Model 3 plus APACHE II, SOFA, mNUTRIC, CCI, Hypertension, sepsis, septic shock, MI, CHF, CPD, DM, VA, MV, RRT, antibiotic, fentanyl, morphine, propofol, midazolam.

Abbreviations: DEX: dexmedetomidine, VAP: ventilator-associated pneumonia, HR: hazard ratio, CI: confidence interval, Ref: reference, ICU: intensive care units, BMI: body mass index, MAP: mean arterial pressure, HB: hemoglobin, PLT: platelet, WBC: white blood cell, AG: anion gap, Scr: serum creatinine, ALT: alanine aminotransferase, APTT: activated partial thromboplastin time, pH: potential of hydrogen, PaCO2: partial pressure of carbon dioxide in arterial blood, PFR: PaO2/FiO2 ratio, APACHE II: Acute Physiology and Chronic Health Evaluation II, SOFA: sequential organ failure assessment, mNUTRIC: Modified Nutrition Risk in Critically ill, CCI: Charlson Comorbidity Index, MI: myocardial infarct, CHF: congestive heart failure, CPD: chronic pulmonary disease, DM: diabetes mellitus, VA: vasopressor agent, MV: mechanical ventilation, RRT: renal replacement treatment.

**FIGURE 2:**
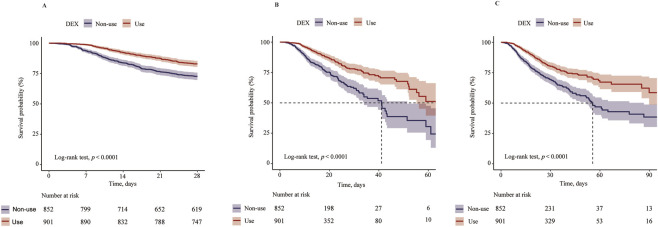
Survival analysis of DEX and non-DEX groups. KM survival curves for the 28-day **(A)**, ICU **(B)** and in-hospital **(C)** mortality among all patients are shown.

KM and adjusted-KM survival curves and log-rank tests further confirmed the benefits of DEX use (Figure 2; Supplementary Figure 1). These findings suggest that the use of DEX provides significant survival benefits for critically ill patients with VAP.

### Sensitivity analysis

#### Propensity score matching analysis

**FIGURE 3:**
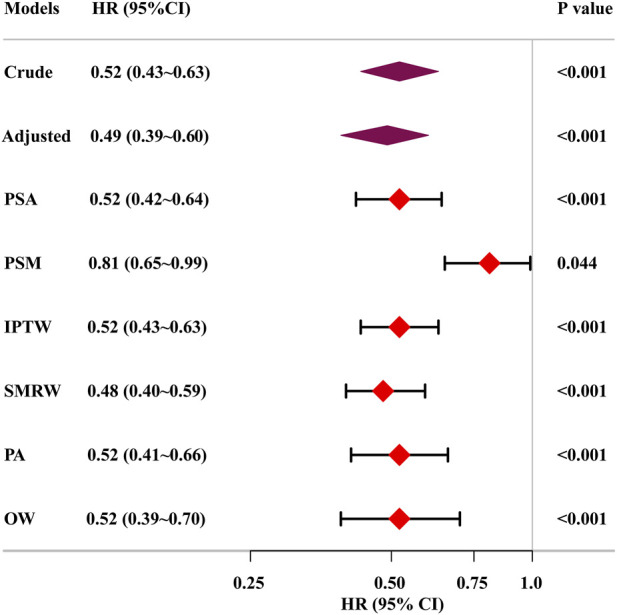
Forest plot displaying HRs from different analytical approaches for 28-day mortality.

A variety of tendency analysis methods such as PSA, PSM, IPTW, SMRW, PA and OW were conducted in the study. The HRs of various methods ranged from 0.48 to 0.81, and all p values were <0.05 (Figure 3).

#### Subgroup analysis

**FIGURE 4:**
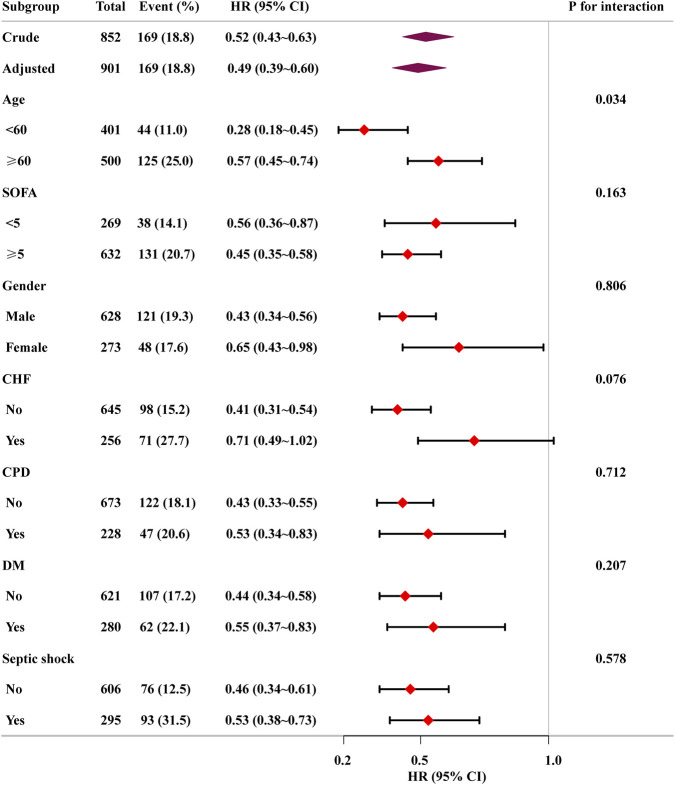
Forest plot of subgroup analysis for 28-day mortality in the DEX group.

Subgroup analysis revealed that DEX use was associated with reduced 28-day mortality in critically ill patients with VAP (Figure 4). No significant interactions were observed between age, gender, SOFA, or comorbidities such as CHF, CPD, DM or CHF, indicating that the observed benefit of DEX use was consistent across these subgroups.

This finding remained consistent across multiple analytical approaches, including univariate and multivariate Cox regression analyses, PSA, PSM, and additional propensity score weighting methods such as IPTW, SMRW, PA and OW. The HRs ranged from 0.48 to 0.81, with all p-values <0.05 (Figure 4), indicating a robust association. Sensitivity analyses (Supplementary Tables 4-6) consistently showed that initiating dexmedetomidine on day 1, within the first 2 days, or within the first 3 days after admitted to the ICU reduced 28-day, in-hospital and ICU mortality in VAP patients (HR 0.50–0.68, all p < 0.05), indicating a robust protective effect regardless of the early-use definition.

## Discussion

In this large retrospective cohort study of 1,753 critically ill patients with VAP, we found that the use of dexmedetomidine (DEX) during ICU stay was associated with a substantially improved survival. Specifically, DEX administration was independently linked to a 46% reduction in 28-day all-cause mortality (HR 0.54, 95% CI 0.43–0.67). This survival benefit was robust, persisting after rigorous multivariable adjustment, propensity score matching, and six alternative balancing algorithms. Beyond the primary outcome, DEX use was also associated with significantly lower ICU and in-hospital mortality. Importantly, the protective effect was consistent across all predefined subgroups, including age, sex, disease severity (SOFA score), and major comorbidities, indicating its broad potential applicability in this vulnerable population. These findings not only reinforce the guideline-recommended use of non-benzodiazepine sedatives but also suggest a specific survival advantage of DEX in the high-risk setting of VAP, prompting a closer examination of its underlying mechanisms.

Although recent multicentre studies indicate that a substantial proportion of mechanically ventilated critically ill adults can be managed without continuous sedative infusions (Strøm et al., 2010; De Bels et al., 2020), our cohort specifically enrolled young male patients with VAP who required deep and sustained sedation to ensure patient-ventilator synchrony and minimize oxygen consumption, a high-risk phenotype in which the risk-benefit profile of sedative agents differs fundamentally from that of the general MV population; in a randomized controlled trial published in JAMA, Pandharipande et al. demonstrated that among MV-ICU patients receiving protocolized (Pandharipande et al., 2007), goal-directed sedation, DEX significantly increased the number of alive-and-delirium-free days compared with propofol or benzodiazepines by activating α_2_-adrenergic rather than γ-aminobutyric acid pathways, thereby preserving spontaneous respiratory drive while attenuating neuro-inflammation—a mechanism consistent with its observed reduction in acute brain dysfunction (Mei et al., 2021). Extending these observations to a septic cohort, our multivariable analysis showed that even after adjustment for illness severity, vasopressor and renal-replacement therapy, DEX remained independently associated with lower 28-day mortality, a survival benefit that is biologically plausible because DEX attenuates systemic inflammation via downregulation of Nuclear Factor kappa-B (NF-κB) and High-Mobility Group Box 1 (HMGB1) signaling, thereby limiting endothelial injury and multi-organ crosstalk in sepsis; large-scale epidemiological studies corroborate these findings, as Wang et al. reported in 2023 that among 15,754 critically ill patients with acute kidney injury, DEX reduced hospital mortality by 38% (HR 0.62, 95% CI 0.55–0.70) and 180-day mortality by 23% (HR 0.77, 95% CI 0.69–0.85) (Wang et al., 2023), while a 2024 propensity-matched retrospective study of 1,075 patients with sepsis-associated encephalopathy showed that DEX was associated with significant reductions in 28-day mortality (HR 0.46, 95% CI 0.35–0.61, p < 0.001) and in-hospital mortality (HR 0.50, 95% CI 0.37–0.67, p < 0.001) (Tang et al., 2024), and the 2013 multicenter randomized DESIRE trial—although underpowered for mortality—observed a numerical decrease in 14-day mortality among septic adults receiving MV who were sedated with DEX (13% vs. 21%, P = 0.16) (Ohta et al., 2020), collectively indicating that DEX not only provides effective sedation in hemodynamically unstable septic patients but also confers a quantifiable survival benefit most likely through combined neuro-protective and systemic anti-inflammatory effects.

The observed survival benefit may stem from DEX’s multimodal effects. First, its α2-adrenergic agonism mitigates sympathetic overdrive, a hallmark of sepsis and critical illness, thereby reducing catecholamine-induced myocardial stress and metabolic dysfunction (Liu et al., 2016; Weerink et al., 2017). Second, DEX preserves respiratory drive and facilitates early extubation, a critical factor in preventing VAP progression and secondary infections. Third, preclinical models suggest DEX enhances bacterial clearance and reduces endothelial injury, potentially interrupting the vicious cycle of infection and organ failure (Liu et al., 2016). Finally, DEX’s unique ability to provide “cooperative sedation” may lower delirium rates, though this warrants further exploration in VAP-specific cohorts. These findings align with preclinical and clinical evidence highlighting DEX’s anti-inflammatory, organ-protective, and delirium-sparing properties, offering a plausible mechanistic basis for its survival benefits in this vulnerable population.

These findings have immediate relevance to ICU practice. While current guidelines recommend DEX or propofol for light sedation (Singer et al., 2016), our findings further demonstrate that DEX reduces 28-day mortality in VAP patients, providing robust evidence to support current guideline recommendations for using DEX to achieve light sedation. Clinicians should prioritize DEX in patients with high inflammatory burden (e.g., elevated CRP, septic shock) or those at risk for prolonged ventilation (Ohta et al., 2020). However, the optimal dosing strategy remains unclear. Notably, the DESIRE trial used lower DEX doses (0.1–0.7 mcg/kg/h) than typical Western protocols, yet still achieved significant anti-inflammatory effects. Future trials should evaluate whether higher doses amplify survival benefits without exacerbating hypotension or bradycardia.

This study has several limitations. First, despite robust statistical adjustments, residual confounding inherent to observational designs cannot be excluded. Unmeasured factors, such as sedation depth or timing of DEX initiation, may influence outcomes. Second, the MIMIC-IV database lacks granular data on DEX dosing, duration, or concurrent analgesic use (e.g., fentanyl), preventing dose-response analyses. Although the MIMIC-IV database records DEX doses, infusion durations, and patient weight, the high interpatient variability in dosing precluded a reliable dose–response analysis. Therefore, we focused on the qualitative association between DEX use and outcomes. Future prospective studies are needed to explore optimal dosing strategies. Third, generalizability may be limited to predominantly White, U.S.-based populations; validation in diverse cohorts is needed.

## Conclusion

DEX administration during ICU stays significantly reduced 28-day mortality in VAP patients with consistent benefit across all subgroups; randomized controlled trials are warranted for validation.

## Data Availability

The original contributions presented in the study are included in the article/Supplementary Material, further inquiries can be directed to the corresponding authors.

## Supplementary material

SUPPLEMENTARY FIGURE 1 Adjusted KM curves for 28-day **(A)**, ICU **(B)**, and in-hospital **(C)** mortality. *Note*: The survival curves have been adjusted for all covariates included in Model 4 of the Cox proportional hazards multivariable analysis.

SUPPLEMENTARY TABLE 1 Selection strategy for variables with multiple measurements.

SUPPLEMENTARY TABLE 2 Variance Inflation Factor (VIF) for variables included in the multivariable Cox regression model.

SUPPLEMENTARY TABLE 3 Missing numbers (%) for covariates.

SUPPLEMENTARY TABLE 4 The associations of DEX-24h use and outcomes in patients with VAP.

SUPPLEMENTARY TABLE 5 The associations of DEX-48h use and outcomes in patients with VAP.

SUPPLEMENTARY TABLE 6 The associations of DEX-72h use and outcomes in patients with VAP.
