# Supplementary material for: Association between dexmedetomidine administration and 28-day mortality in critically ill patients with ventilator-associated pneumonia
Source: Front Pharmacol. 2026 Jun 25;17:1785115. doi: 10.3389/fphar.2026.1785115 (PMC13347083; doi:10.3389/fphar.2026.1785115)
Supplement: Supplementary file 1 [file Table1.docx]

**Supplementary Table 1.** Selection strategy for variables with multiple measurements.

| Variables | Details |
| --- | --- |
| Heart rate | Record the **highest** value for 24 hours of ICU admission |
| MAP | Record the **lowest** value for 24 hours of ICU admission |
| Respiratory rate | Record the **highest** value for 24 hours of ICU admission |
| WBC | Record the **highest** value for 24 hours of ICU admission |
| HB | Record the **lowest** value for 24 hours of ICU admission |
| PLT | Record the **lowest** value for 24 hours of ICU admission |
| pH | Record the **lowest** value for 24 hours of ICU admission |
| PFR | Record the **lowest** value for 24 hours of ICU admission |
| PaCO_2_ | Record the **highest** value for 24 hours of ICU admission |
| Bicarbonate | Record the **lowest** value for 24 hours of ICU admission |
| AG | Record the **highest** value for 24 hours of ICU admission |
| Lactate | Record the **highest** value for 24 hours of ICU admission |
| SCr | Record the **highest** value for 24 hours of ICU admission |
| ALT | Record the **highest** value for 24 hours of ICU admission |
| APTT | Record the **highest** value for 24 hours of ICU admission |
| Potassium | Record the **highest** value for 24 hours of ICU admission |
| Sodium | Record the **highest** value for 24 hours of ICU admission |
| Calcium | Record the **lowest** value for 24 hours of ICU admission |

***Abbreviations*:**

ICU: intensive care unit, MAP: mean arterial pressure, WBC: white blood cell, HB: hemoglobin, PLT: platelet, pH: potential hydrogen, PFR: PaO_2_/FiO_2_ ratio, PaCO_2_: partial pressure of carbon dioxide of arterial blood, AG: anion gap, SCr: serum creatinine, ALT: alanine transaminase, APTT: activated partial thromboplastin time.
