# Supplementary figures and images for: Association between dexmedetomidine administration and 28-day mortality in critically ill patients with ventilator-associated pneumonia
Source: Front Pharmacol. 2026 Jun 25;17:1785115. doi: 10.3389/fphar.2026.1785115 (PMC13347083; doi:10.3389/fphar.2026.1785115)

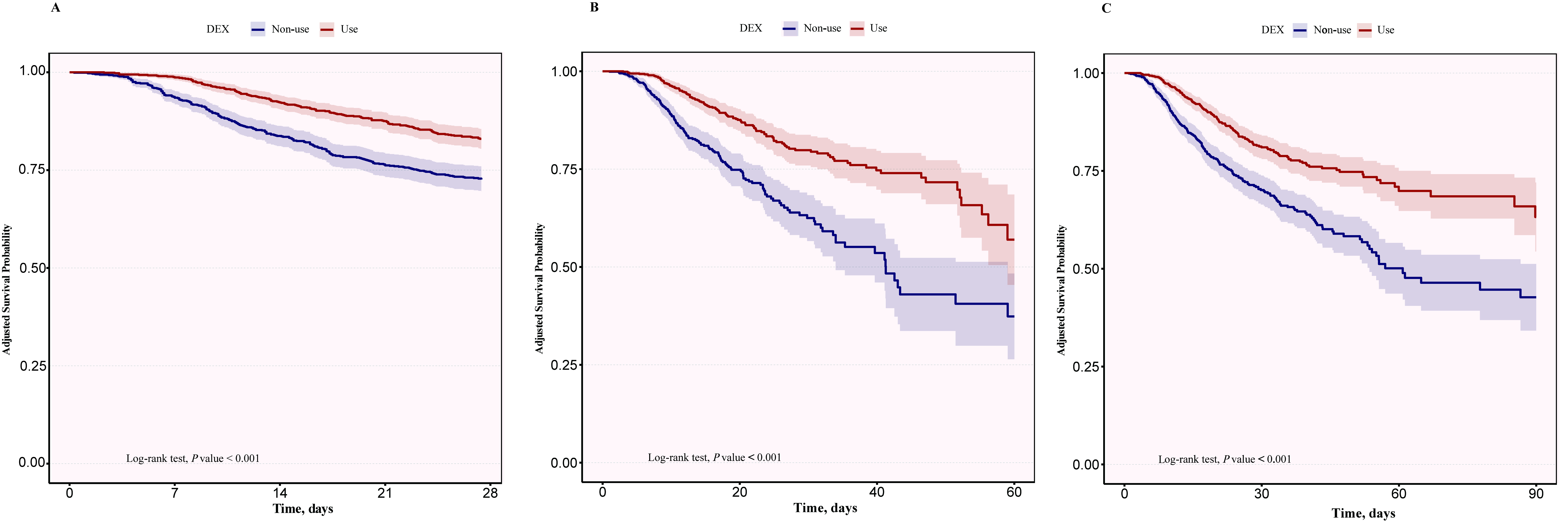

Supplement: Supplementary file 2 [file Image1.tif]
