# Supplementary material for: Association between dexmedetomidine administration and 28-day mortality in critically ill patients with ventilator-associated pneumonia
Source: Front Pharmacol. 2026 Jun 25;17:1785115. doi: 10.3389/fphar.2026.1785115 (PMC13347083; doi:10.3389/fphar.2026.1785115)
Supplement: Supplementary file 3 [file Table2.docx]

**Supplementary Table 2.** Variance Inflation Factor (VIF) for variables included in the multivariable Cox regression model.

| Variable | VIF |
| --- | --- |
| Gender | 1.187 |
| Age | 2.286 |
| Race | 1.087 |
| BMI | 1.301 |
| Heart rate | 1.143 |
| Respiratory rate | 1.220 |
| MAP | 1.244 |
| Hemoglobin | 1.380 |
| AG | 2.599 |
| SCr | 2.083 |
| ALT | 1.230 |
| APTT | 1.272 |
| Potassium | 1.460 |
| Sodium | 1.191 |
| Calcium | 1.463 |
| pH | 5.561 |
| PaCO_2_ | 4.148 |
| PFR | 1.786 |
| Lactate | 2.382 |
| Bicarbonate | 4.890 |
| APACHE II | 4.591 |
| SOFA | 4.380 |
| mNUTRIC | 6.189 |
| Hypertension | 1.232 |
| CCI | 2.218 |
| Sepsis | 1.121 |
| Sepsis shock | 1.265 |
| MI | 1.310 |
| CHF | 1.400 |
| CPD | 1.177 |
| DM | 1.357 |
| VA | 1.746 |
| MV | 2.238 |
| RRT | 1.365 |
| Fentanyl | 1.800 |
| Midazolam | 1.509 |
| Morphine | 1.121 |
| Propofol | 1.766 |
| Antibiotic | 1.246 |
| Platelets | 1.432 |
| WBC | 1.110 |

***Abbreviations:***

BMI: body mass index, MAP: mean arterial pressure, AG: anion gap, SCr: serum creatinine, ALT: alanine aminotransferase, APTT: activated partial thromboplastin time, PaCO₂: partial pressure of carbon dioxide, PFR: PaO₂/FiO₂ ratio, APACHE II: Acute Physiology and Chronic Health Evaluation II, SOFA: Sequential Organ Failure Assessment, mNUTRIC: modified Nutrition Risk in Critically Ill, CCI: Charlson Comorbidity Index, MI: myocardial infarct, CHF: congestive heart failure, CPD: chronic pulmonary disease, DM: diabetes mellitus, VA: vasoactive drugs, MV: mechanical ventilation, RRT: renal replacement therapy, WBC: white blood cell count.
