# Supplementary material for: Association between dexmedetomidine administration and 28-day mortality in critically ill patients with ventilator-associated pneumonia
Source: Front Pharmacol. 2026 Jun 25;17:1785115. doi: 10.3389/fphar.2026.1785115 (PMC13347083; doi:10.3389/fphar.2026.1785115)
Supplement: Supplementary file 4 [file Table5.docx]

**Supplementary Table 5.** The associations of DEX-48h use and outcomes in patients with VAP

| **Variable** | **n.total** | **n.event %** | **Model 1** | **Model 2** | **Model 3** | **Model 4** |
| --- | --- | --- | --- | --- | --- | --- |
|  |  |  | **HR (95% CI)** | **HR (95% CI)** | **HR (95% CI)** | **HR (95% CI)** |
| **In-hospital mortality** |  |  |  |  |  |  |
| DEX-48h (non-use) | 1417 | 365 (25.8) | 1(Ref) | 1(Ref) | 1(Ref) | 1(Ref) |
| DEX-48h (use) | 336 | 58 (17.3) | 0.65 (0.49~0.86) | 0.63 (0.48~0.84) | 0.64 (0.49~0.85) | 0.61 (0.46~0.81) |
| *P* value |  |  | 0.002 | 0.001 | 0.002 | 0.001 |
| **ICU mortality** |  |  |  |  |  |  |
| DEX-48h (non-use) | 1417 | 260 (18.3) | 1(Ref) | 1(Ref) | 1(Ref) | 1(Ref) |
| DEX-48h (use) | 336 | 41 (12.2) | 0.63 (0.46~0.88) | 0.60 (0.43~0.84) | 0.62 (0.44~0.86) | 0.58 (0.42~0.82) |
| *P* value |  |  | 0.007 | 0.003 | 0.004 | 0.002 |
| **28-day mortality** |  |  |  |  |  |  |
| DEX-48h (non-use) | 1417 | 333 (23.5) | 1(Ref) | 1(Ref) | 1(Ref) | 1(Ref) |
| DEX-48h (use) | 336 | 54 (16.1) | 0.65 (0.48~0.86) | 0.66 (0.50~0.88) | 0.66 (0.49~0.88) | 0.62 (0.46~0.83) |
| *P* value |  |  | 0.003 | 0.005 | 0.005 | 0.001 |

***Notes***:

Model 1: Unadjusted;

Model 2: Adjusted for gender, age, race, BMI;

Model 3: Model 2 plus heart rate, respiratory rate, MAP, HB, PLT, WBC, AG, SCr, ALT, APTT, potassium, sodium, calcium, pH, PaCO_2_, PFR, lactate,

bicarbonate;

Model 4: Model 3 plus APACHE II, SOFA, mNUTRIC, CCI, hypertension, sepsis, septic shock, MI, CHF, CPD, DM, VA, MV, RRT, antibiotic, fentanyl, morphine, propofol, midazolam.

***Abbreviations*:** DEX, dexmedetomidine, VAP: Ventilator-associated pneumonia, HR: hazard ratio, CI: confidence interval, Ref: reference, BMI, body mass index, MAP: mean arterial pressure, HB: hemoglobin, PLT: platelet, WBC: white blood cell, AG: anion gap, SCr: serum creatinine, ALT: alanine aminotransferase, APTT: activated partial thromboplastin time, pH: potential of hydrogen, PaCO_2_: partial pressure of carbon dioxide in arterial blood, PFR: PaO_2_/FiO_2_ ratio, APACHE II: Acute Physiology and Chronic Health Evaluation II, SOFA: sequential organ failure assessment, mNUTRIC: Modified Nutrition Risk in Critically ill, CCI: Charlson Comorbidity Index, MI: myocardial infarct, CHF: congestive heart failure, CPD: chronic pulmonary disease, DM: diabetes mellitus, VA: vasopressor agent, MV: mechanical ventilation, RRT: renal replacement treatment.
