# Supplementary material for: Association between dexmedetomidine administration and 28-day mortality in critically ill patients with ventilator-associated pneumonia
Source: Front Pharmacol. 2026 Jun 25;17:1785115. doi: 10.3389/fphar.2026.1785115 (PMC13347083; doi:10.3389/fphar.2026.1785115)
Supplement: Supplementary file 5 [file Table3.docx]

**Supplementary Table 3.** Missing numbers (%) for covariates.

| **Variables** | **Miss frequency** | **Miss percentage %** |
| --- | --- | --- |
| **General characteristics** |  |  |
| Age | 0 | 0 |
| Gender | 0 | 0 |
| Race | 0 | 0 |
| BMI | 220 | 12.55 |
| **Vital signs** |  |  |
| Heart rate | 1 | 0.06 |
| Respiratory rate | 2 | 0.11 |
| MAP | 1 | 0.06 |
| **Laboratory parameters** |  |  |
| WBC | 10 | 0.57 |
| HB | 10 | 0.57 |
| PLT | 10 | 0.57 |
| AG | 13 | 0.74 |
| Bicarbonate | 12 | 0.68 |
| SCr | 10 | 0.57 |
| ALT | 461 | 26.30 |
| Sodium | 10 | 0.57 |
| Calcium | 40 | 2.28 |
| Potassium | 11 | 0.63 |
| PaCO_2_ | 181 | 10.33 |
| PFR | 305 | 17.40 |
| pH | 181 | 10.33 |
| Lactate | 404 | 23.05 |
| APTT | 73 | 4.16 |
| **Disease severity scores** |  |  |
| APACHE II | 0 | 0 |
| SOFA | 0 | 0 |
| CCI | 0 | 0 |
| mNUTRIC | 0 | 0 |
| **Comorbidities** |  |  |
| MI | 0 | 0 |
| CHF | 0 | 0 |
| CPD | 0 | 0 |
| Hypertension | 0 | 0 |
| DM | 0 | 0 |
| Sepsis | 0 | 0 |
| Septic shock | 0 | 0 |
| **Medication or procedures** |  |  |
| Fentanyl | 0 | 0 |
| Midazolam | 0 | 0 |
| Morphine | 0 | 0 |
| Propofol | 0 | 0 |
| Antibiotic | 0 | 0 |
| VA | 0 | 0 |
| MV | 0 | 0 |
| RRT | 0 | 0 |

***Abbreviations*:** BMI: body mass index, MAP: mean arterial pressure, WBC: white blood cell, HB: hemoglobin, PLT: platelet, AG: anion gap, SCr: serum creatinine, ALT, alanine aminotransferase, PaCO_2_: partial pressure of carbon dioxide in arterial blood, PFR: PaO_2_/FiO_2_ ratio, pH: potential of hydrogen, APTT: activated partial thromboplastin time, APACHE II: Acute Physiology and Chronic Health Evaluation II, SOFA: sequential organ failure assessment, CCI: Charlson Comorbidity Index, mNUTRIC: Modified Nutrition Risk in Critically ill, MI: myocardial infarction, CHF: congestive heart failure, CPD: chronic pulmonary disease, DM: diabetes mellitus, VA: vasopressor agent, MV: mechanical ventilation, RRT: renal replacement treatment.
